# Electrochemical performance of ZnO-coated Li_4_Ti_5_O_12_ composite electrodes for lithium-ion batteries with the voltage ranging from 3 to 0.01 V

**DOI:** 10.1098/rsos.180762

**Published:** 2018-10-31

**Authors:** Ying Wang, Ya Ren, Xinyi Dai, Xiao Yan, Bixiong Huang, Jingze Li

**Affiliations:** 1 School of Mechanical and Automotive Science, Shanghai University of Engineering Science, Shanghai 201620, People’s Republic of China; 2 College of Materials and Metallurgy, Guizhou University, Guiyang 550025, People’s Republic of China; 3 School of Microelectronics and Solid-state Electronics, University of Electronic Science and Technology of China, Chengdu 610054, People’s Republic of China

**Keywords:** magnetron sputtering, ZnO, Li_4_Ti_5_O_12_, anode, lithium-ion battery

## Abstract

Oxide is widely used in modifying cathode and anode materials for lithium-ion batteries. In this work, a facile method of radio magnetron sputtering is introduced to deposit a thin film on Li_4_Ti_5_O_12_ composite electrodes. The pristine and modified Li_4_Ti_5_O_12_ electrodes are characterized at an extended voltage range of 3–0.01 V. The reversible capacity reaches a high level of 286 mAh g^−1^, which is a little less than its theoretical capacity (293 mAh g^−1^). Electrodes modified by ZnO thin films with various thickness show elevated rate capability and improved cycle performance.

## 1. Introduction

Spinel Li_4_Ti_5_O_12_ has been considered one of the most promising anodes for high power lithium-ion batteries [1,2]. Surface coating [3–6], cation doping [7–10] and making composites [11–13] have been intensively studied and demonstrated to be three main methods to modify Li_4_Ti_5_O_12_ powders effectively in the voltage range of 3–1 V. Most recently, it becomes more attractive with the greatly improved theoretical capacity (293 mAh g^−1^) in the extended voltage range of 3–0.01 V [14–16]. A small amount of research has been conducted to improve the electrochemical performance by cation doping [17–21] and making composites [22,23]. However, the voltage extension to such a low potential will inevitably induce the formation of solid electrolyte interface (SEI) film on the surface of acetylene black and pristine Li_4_Ti_5_O_12_ as well as modified Li_4_Ti_5_O_12_ particles. So it is difficult for powder modification to provide a solution to avoid the block between the particles of Li_4_Ti_5_O_12_ and acetylene black hindering the electron transportation. Surface coating based on electrodes has demonstrated a potential method to modify LiCoO_2_ [24–29] and LiFePO_4_ [30], which introduce an artificial SEI film for the electrode and keeps the integrity of electronic conducting network. Oxides are usually chosen to be the coating materials, among which zinc oxide has attracted a lot of attention in optoelectronic devices, solar cells and gas sensors. For lithium-ion batteries, it is often used as HF scavengers to modify cathode powders. Apart from these applications, recent researches have demonstrated that ZnO can be reacted with lithium reversibly [31,32]. The electrochemical process can be expressed as follows:

1.1 $$\text{ZnO}+\text{2Li}\to \text{Zn}+{\text{Li}}_{2}\text{O}$$

and

1.2 Zn+Li↔LiZn.

Zn and lithium-zinc alloys are good electronic conductors. Li_2_O is the framework to accommodate lithium-zinc alloys. The two-step reaction releases a theoretical capacity as high as 978 mAh g^−1^ [33]. This particular characteristic gives rise to an intersection conducting network for electrons and lithium ions, which declares its potential use in surface modification of anode electrodes.

In this work, a simple radio frequency magnetron sputtering method was employed to deposit ZnO thin films on conventionally fabricated spinel Li_4_Ti_5_O_12_ composite electrodes. The thin layer evolved from the reaction between Li and ZnO during discharge and charge process consisted of Zn-Li alloy and Li_2_O which formed a hybrid conducting network between the active material and conductive additive particles. At the same time, an artificial layer protecting the electrodes from SEI formation on individual particles took shape as well. A resultant fluency electron transportation was then achieved. The rate performance and cycle stability were improved as expected.

## 2. Experimental procedure

### 2.1. Preparation of Li_4_Ti_5_O_12_ powder and ZnO-coated composite electrodes

To prepare Li_4_Ti_5_O_12_ powder, Li_2_CO_3_ (industrial grade) and anatase TiO_2_ (AR, 100 nm) were adequately mixed in a planetary ball mill, dried at 80°C in the air and synthesized in a tube furnace at 750°C in air atmosphere, in sequence. The details have been reported in previous work [13].

A conventional doctor blade method was employed to prepare Li_4_Ti_5_O_12_ composite electrodes. The weight ratio of active material (Li_4_Ti_5_O_12_), conducting addictive (acetylene black) and binder (PVDF) was 8 : 1 : 1. The mixed precursor was spread on copper foil and dried for 12 h for later use. The average mass loading was larger than 1.3 mg cm^−2^.

A radio frequency magnetron sputtering system was employed to deposited ZnO thin layer. ZnO target (99.99%, JiagXi Hai Te Advanced Material Co., Ltd) was commercially available. The as-prepared electrodes were transferred into the chamber and located in the substrate position. The chamber was vacuumized to a base pressure of 6.0 × 10^−4^ Pa. Thereafter the substrate was rotated at the speed of 10 r.p.m. and heated to 120°C, which was held throughout the deposition process. The heating time before sputtering was set to 1 h to help evacuating the residual air. Argon (99.999%) was introduced as the working gas. The sputtering parameter was set as follows: substrate-to-target distance, 5 cm; working pressure, 0.5 Pa; gas flow, 50 SCCM (standard cubic centimetres per minute); RF power 80 W; pre-sputtering time, 5 min. The deposition time varied from 1 to 10 min. Samples deposited for 1 min, 5 min and 10 min was designated as LTOZnO01, LTOZnO05 and LTOZnO10, respectively.

### 2.2. Physical and electrochemical characterization

X-ray diffraction (XRD, X’pert pro MPD, Cu Kα) was used to collect phase information of the pristine and coated electrodes in the scan range of 10–85° with a step size of 0.03° s^−1^. The corresponding morphology and energy dispersion spectrum were detected by field emission scanning electron microscope (FE-SEM, Hitachi, S3400N).

Two-electrode laboratory cells were assembled to test the electrochemical performance. The working electrodes were prepared by cutting the coated electrodes into round pieces (*ϕ* = 1 cm) and put into an argon-circulated glove box (argon, 99.99%, O_2_ < 5 ppm; H_2_O < 5 ppm) for cell assembly. Lithium foil, Celgard microporous polypropylene and 1 M LiPF6 solution (solvent, a mixture of ethylene carbonate (EC) and diethyl carbonate (DEC) (1 : 1 in volume)) were used as counter electrode, separator and electrolyte, respectively.

Galvanostatic discharge-charge data were collected in an extended voltage range of 3–0.01 V by a LAND series battery testing system (CT2001A, LAND Electronic Co.). The current density varied from 35 to 880 mA g^−1^. Cyclic voltammetry (CV) profiles (0.1 mV s^−1^, 3–0.01 V) and electrochemical impedance spectra (EIS) plots (1.563 V, 0.1 Hz–1 MHz) were derived from measurements carried out on an electrochemical analyser (Solartron Model 1287/1260A; Solartron Analytical).

## 3. Results and discussion

### 3.1. Structure and morphology of pristine and ZnO-coated Li_4_Ti_5_O_12_ composite electrodes

**Figure 1.**
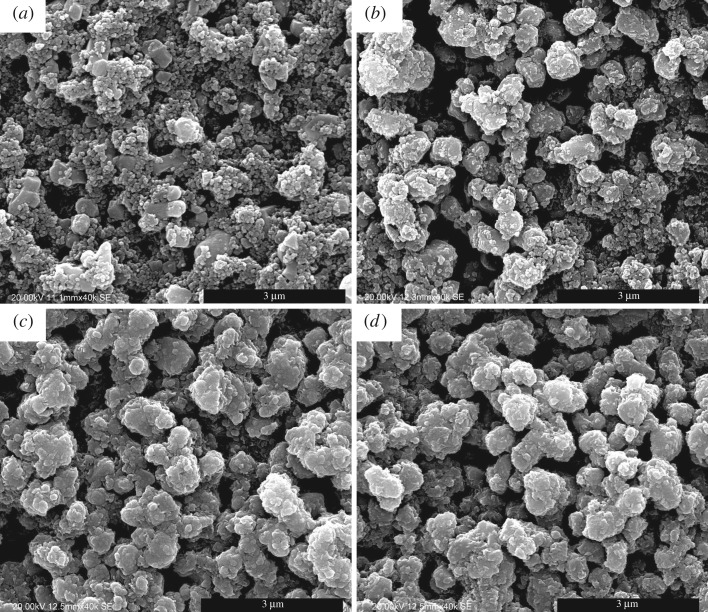
SEM images of pristine electrode (*a*) LTOZnO00 and ZnO-coated Li_4_Ti_5_O_12_ composite electrodes ((*b*) LTOZnO01 (1 min), (*c*) LTOZnO05 (5 min) and (*d*) LTOZnO10 (10 min)).

The morphology information of pristine electrode (LTOZnO00) and ZnO-coated electrodes (LTOZnO01, 1 min; LTOZnO05, 5 min; LTOZnO10, 10 min) is delivered in figure 1. As shown in figure 1*a*, it is easy to distinguish the larger Li_4_Ti_5_O_12_ particles from the smaller acetylene carbon particles. The particles of Li_4_Ti_5_O_12_ disperse homogeneously in the matrix of those of acetylene carbon, which ensure a fully exerted electrochemical performance. A not very prominent but discernable difference can be seen between the morphology of pristine and 1 min ZnO-coated Li_4_Ti_5_O_12_ composite electrodes (shown in figure 1*b*). The difference becomes much more pronounced for the sample deposited for 5 min (shown in figure 1*c*). There is no obvious morphology alteration when the sputtering time is lengthened to 10 min, which demonstrates that a relatively intact cover layer has been formed for LTOZnO05. All the ZnO layers deposited on the composite electrodes are composed of very tiny particles.

**Figure 2.**
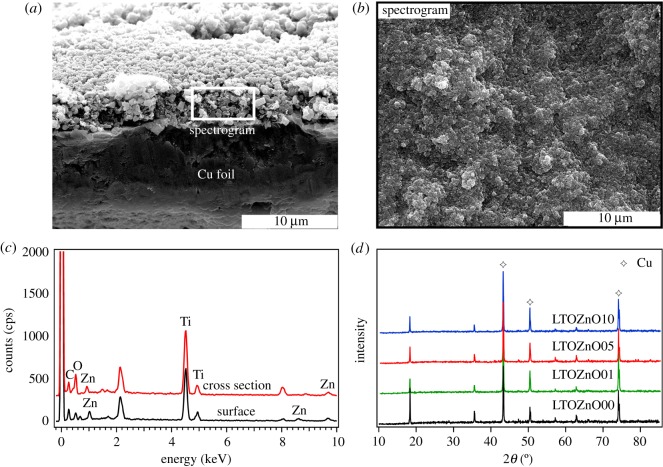
The selected cross section (*a*) and surface (*b*) areas for EDS tests, (*c*) the EDS results of ZnO (5 min) coated Li_4_Ti_5_O_12_ electrodes, (*d*) XRD patterns of pristine electrode LTOZnO00 and ZnO-coated Li_4_Ti_5_O_12_ composite electrodes (LTOZnO01 (1 min), LTOZnO05 (5 min) and LTOZnO10 (10 min).

Energy dispersive spectroscopy (EDS) is carried out to further evaluate the composition of the cross section and the surface for ZnO-coated Li_4_Ti_5_O_12_ electrodes, as shown in figure 2*a*–*c*. The sputtered ZnO thin layer can not be clearly distinguished from the pristine Li_4_Ti_5_O_12_ electrode for its small thickness. To estimate the thickness of ZnO film, pure ZnO film is deposited on a bare Cu foil for 20 min. Given the density of the film, 5.606 g cm^−3^ (the same as the bulk density), the thickness of ZnO thin layer (5 min) is estimated to be 47.5 nm, which is so small that it is difficult to identify in figure 2*a*. The energy of 0.906, 1.012, 8.639 and 9.572 keV can be ascribed to the existence of Zn element. The mass ratio of Zn is very small (less than 5%) for the selected cross-sectional area. The mass ratio of Zn reaches 6.94% (versus the total mass of C, O, Ti and Zn) for the surface area. The XRD patterns of pristine and ZnO-coated Li_4_Ti_5_O_12_ composite electrodes are shown in figure 2*d*. The X-ray diffraction peaks marked with asterisk match the peaks listed in PDF#04-0836, which belong to copper. The diffraction peaks of pristine electrodes are in good agreement with those of as-synthesized powder which have been reported in the literature [13]. No discriminable sign of ZnO diffraction peaks is observed for coated electrodes, which attributes to the tiny particles seen in SEM images or the poor crystallinity. The coherence of the diffraction peaks of pristine and ZnO-coated Li_4_Ti_5_O_12_ composite electrodes illustrates that no side-effect has been exerted on the structure of Li_4_Ti_5_O_12_ material.

### 3.2. Electrochemical performance of pristine and ZnO-coated Li_4_Ti_5_O_12_ composite electrodes

**Figure 3.**
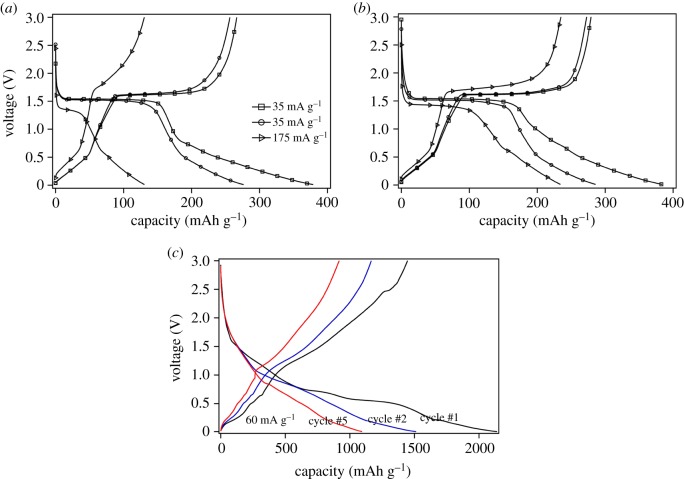
Discharge–charge profiles of (*a*) pristine electrode LTOZnO00, (*b*) coated Li_4_Ti_5_O_12_ composite electrodes LTOZnO05 (5 min) and (*c*) sputtered ZnO (20 min) film on Cu foil.

The discharge–charge profiles of pristine and ZnO-coated Li_4_Ti_5_O_12_ composite electrodes are shown in figure 3*a*,*b*. The shapes of the discharge–charge profile for the first cycle are similar, containing two voltage plateaus. The plateau at the voltage of about 1.5 V is corresponding to the coexisting of Li_4_Ti_5_O_12_ and Li_7_Ti_5_O_12_ phases. The plateau at about 0.7 V is indicative of SEI film formation. The discharge capacity of the first cycle at the voltage of 1 V is 171 and 198 mAh g^−1^ for pristine and ZnO-coated Li_4_Ti_5_O_12_ (5 min) electrodes, respectively. The corresponding capacities decrease to 162 and 174 mAh g^−1^, respectively, at the second cycle. The capacity increment of ZnO-coated Li_4_Ti_5_O_12_ (5 min) electrode at 1 V is ascribed to conducting network evolved from the coating layer rather than contributed by ZnO material itself, for the plateau of ZnO anode reported is below 1 V [32]. Similar results has been reported for epitaxial grown Li_4_Ti_5_O_12_ thin films by R. Kanno group [34]. When the batteries are further discharged to 0.01 V, the discharge capacities increase to 378 mAh g^−1^ and 383 mAh g^−1^ for the first cycle, which are much higher than the reported capacity of 293 mAh g^−1^ [14]. The increased initial capacity is contributed by ZnO thin layer and the SEI formation. As shown in figure 3*c*, the specific capacity of ZnO thin layer deposited for 20 min is more than 2000 mAh g^−1^ in the first discharge–charge cycle. The capacity decreases rapidly to 1083 mAh g^−1^ at the fifth cycle, demonstrating bad cycle performance. The lost capacity of the first cycle is caused by the irreversible reaction with a resultant of Li_2_O (shown in equation (1.1)) and SEI film. Combined with Zn or LiZn alloy, a layer of hybrid conducting network is formed which guarantees better rate performance for ZnO-modified electrodes. The following capacity loss is due to the volume expansion during the formation of LiZn alloy. The discharge capacities of pristine and ZnO-coated Li_4_Ti_5_O_12_ composite electrodes decrease to 276 and 286 mAh g^−1^ (3–0.01 V) for the second cycle. The ratios of discharge capacity at a voltage range of 3–1 V to that of 3–0.01 V are different for pristine and modified samples. ZnO layer coated on the active material particles is speculated to have an impact on the SEI film formation at the interface between active material, acetylene black and electrolyte. The polarization of pristine electrode (LTOZnO00) grows sharply accompanying with dramatic capacity decline when the discharge–charge current density is elevated to 175 mA g^−1^. By contrast, this trend slows down significantly for ZnO-coated electrodes. The capacity decline between 1 and 0.01 V is smaller than that between 3 and 1 V, which is speculated that it is an energetically favoured lithium storage process rather than a kinetic one that dominates in the voltage range of 1–0.01 V [10,35].

**Figure 4.**
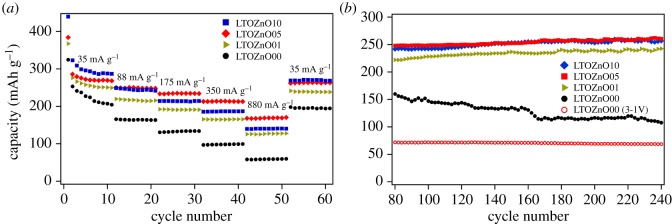
(*a*) Rate performance and (*b*) cycle capability of pristine electrode LTOZnO00 and coated Li_4_Ti_5_O_12_ composite electrodes (LTOZnO01 (1 min), LTOZnO05 (5 min) and LTOZnO10 (10 min)).

The rate performance of pristine and ZnO-coated Li_4_Ti_5_O_12_ is shown in figure 4*a*. Evidently, the capacity of the modified Li_4_Ti_5_O_12_ electrodes increases with the thickening of ZnO coating layers for the first 10 cycles, which can be partly attributed to the capacity of ZnO anode material. When the discharge–charge current is elevated to 88 mA g^−1^, the capacity experiences a decrease for all the pristine and modified samples. However, the capacity decrease of all the Li_4_Ti_5_O_12_ electrodes coated with ZnO layer is much less than that of pristine electrodes. When the discharge–charge current is further increased, the trend is maintained. The result is indicative that it is the hybrid thin layer evolved by the interaction of ZnO-coating layer and lithium other than ZnO material itself that contributes to the enhancement of the rate performance. When the sputtering time is 1 min, the thickness is estimated to be less than 10 nm, which is not thick enough to form hybrid network with good conductivity. As ZnO film grows thicker, a better conducting network can be possibly formed. However, if the ZnO film becomes thicker and thicker (e.g. 100 nm), cracks will appear during the dealloying process after the volume expansion when alloying reaction occurs. The cracks will deteriorate the integrity of the conducting network. In this work, best rate performance is achieved with the electrodes coated with film deposited for 5 min indicating an estimated 47.5 nm ZnO layer can evolve into a good conducting network.

The capacities of Li_4_Ti_5_O_12_ composite electrode modified by 5 min-sputtering ZnO coating layer are 270, 250, 234, 212 and 168 mAh g^−1^ when the discharge–charge currents are 35, 88, 175, 350 and 875 mA g^−1^, respectively. When the discharge–charge current is reduced 35 mA g^−1^, the remaining capacities of the pristine and ZnO-coated (5 min) Li_4_Ti_5_O_12_ composite electrodes are 198 and 261 mAh g^−1^. To evaluated the cycle stability of the pristine and ZnO-coated Li_4_Ti_5_O_12_ composite electrodes, the batteries are further tested with a discharge–charge current of 88 mA g^−1^ for another 160 cycles. All the modified samples show excellent cycle stability (as shown in figure 4*b*). While the cycle performance of the pristine electrode experiences a quicker decrease than the modified electrodes when discharge–charged at the potential range of 3–0.01 V, good cycle stability is achieved when the potential range is set between 3 and 1 V, even the rate is as high as 10 C.

**Figure 5.**
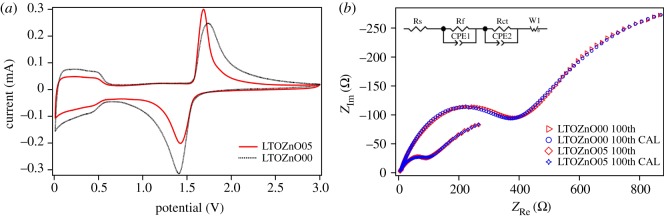
The electrochemical behaviour of the pristine (LTOZnO00) and ZnO-coated Li_4_Ti_5_O_12_ (LTOZnO05 (5 min)) composite electrodes. (*a*) CV curves at the scan rate of 0.1 mV s^−1^ and (*b*) electrochemical impedance spectroscopy collected after galvanostatic discharge–charged for 100 cycles.

The electrochemical behaviour of the pristine and ZnO-coated Li_4_Ti_5_O_12_ composite electrodes is investigated by cyclic voltammograms (CV) at a scan rate of 0.1 mV s^−1^ between 0 and 3 V and electrochemical impedance spectroscopy after galvanostatic discharge–charged for 100 cycles. The CV curves of the pristine and modified electrodes are shown in figure 5*a*. It can be concluded that there are two pairs of redox peaks for all the samples. One of the reduction peaks locates at 1.404 and 1.426 V, respectively, in the third cycle for LTOZnO00 and LTOZnO05. Meanwhile the corresponding oxidation peaks, 1.738 and 1.686 V, are detected for LTOZnO00 and LTOZnO05. The polarization of this pair of peaks is reduced by the ZnO coating layer. In addition, the redox peaks of ZnO-modified electrodes are sharper and show better-defined splitting than pristine electrodes, indicating better reversibility. The other pair of redox peaks are below 0.6 V, indicating the phase transformation from rock-salt [Li_2_]_16*c*_[Li_1/3_Ti_5/3_]_16*d*_[O_4_]_32*e*_ phase to quasi-rock-salt Li_8*a*_[Li_2_]_16*c*_[Li_1/3_Ti_5/3_]_16*d*_[O_4_]_32*e*_ phase.

The measured and calculated Nyquist plots are shown in figure 5*b*. The calculated data agree with the measured data very well when the inserted equivalent circuit (as shown in figure 5*b*) is employed to fit the data. Both the measured plots consist of two compressed semicircles and an inconspicuous oblique tail. The high-frequency semicircle is related to the resistance of SEI film formed on the electrode at a low voltage of 0.7 V. The intermediate frequency semicircle is attributed to the charge transfer resistance between the interface of active materials and electrolyte. The oblique line is associated with the Li-ion diffusion within the active materials. The fitted values of Rs of the pristine and ZnO-coated Li_4_Ti_5_O_12_ composite electrodes are less than 5 Ω. The fitted value of the SEI film resistance (Rf) of ZnO-coated Li_4_Ti_5_O_12_ (5 min) composite electrodes is 101 Ω, which is much smaller than 364 Ω for the pristine electrode. The fitted values of charge transfer resistances (Rct) are 224 and 1019 Ω for pristine and ZnO-coated Li_4_Ti_5_O_12_ (5 min) composite electrodes, respectively. The reduced SEI film resistance and charge transfer resistance ensured elevated rate performance and stable cycle performance.

## 4. Conclusion

In summary, Li_4_Ti_5_O_12_ composite electrodes were successfully coated with ZnO thin films via a radio frequency magnetron sputtering method. The thickness of ZnO films was tailored by altering depositing time. A mixture layer of Zn-Li alloy and Li_2_O produced by ZnO coating film during the discharge process was suggested to improve the interface between electrode and electrolyte. As a result, the rate performance and cycle stability were greatly improved by ZnO coating layer.

## Data accessibility

Our data are deposited at Dryad: https://doi.org/10.5061/dryad.cp4008d [36].
